# The renin angiotensin aldosterone system

**DOI:** 10.1007/s00424-024-02908-1

**Published:** 2024-01-17

**Authors:** Hannah Triebel, Hayo Castrop

**Affiliations:** Institute of Physiology, University of Regensburg, Universitätsstr. 31, 93040 Regensburg, Germany

**Keywords:** Renin, Angiotensin, Volume homeostasis, Vascular resistance, Potassium

## Abstract

In this review, we will cover (i) the proteolytic cascade of the RAAS, (ii) its regulation by multiple feedback-controlled parameters, and (iii) the major effects of the RAAS. For the effects of the RAAS, we focus on the role of the RAAS in the regulation of volume homeostasis and vascular tone, as major determinants of arterial blood pressure.

## Introduction

The renin angiotensin aldosterone system (RAAS) is a hormonal system for which the primary effector is angiotensin 2, which is derived from stepwise proteolytic cleavage of angiotensinogen. Angiotensin 2, in turn, is a stimulator of aldosterone synthesis. Consequently, angiotensin 2 and aldosterone are the primary endpoints of the hormonal system. During recent decades, our knowledge regarding the RAAS has expanded considerably, and novel functional aspects have been added consistently. Nevertheless, it appears reasonable to state that the primary function of the RAAS is the regulation of arterial blood pressure.

Arterial blood pressure is determined by the cardiac output per time and the total vascular resistance. Consequently, arterial blood pressure is a function of blood volume, respective of the extracellular volume. The long-term homoeostasis of the extracellular volume is regulated by a balanced intake of salt and water and the concomitant excretion of the same amount of salt and water, with the kidney accounting for the bulk of salt and water elimination. Angiotensin 2 influences the intake of salt and water by triggering feelings of thirst and salt appetite, which, given an adequate availability of salt and water, leads to behavioral changes, such as drinking and salt seeking. On the side of balanced excretion, angiotensin 2 reduces body salt and water losses by direct and indirect renal effects. The direct effects comprise changes in the glomerular filtration rate (GFR) and the modulation of tubular salt reabsorption. The indirect effect on renal salt and water handling includes the formation of aldosterone, which directly promotes renal Na^+^ conservation. Aldosterone-dependent Na^+^ conservation, however, is inevitably linked to renal K^+^ losses. Consequently, body Na^+^ and K^+^ homeostasis is interlinked, which limits the regulatory range for each of the variables.

In terms of vascular resistance, the second determinant of arterial blood pressure, angiotensin 2 is one of the most potent vasoconstrictors of the body, causing blood pressure to stabilize. Whereas the vascular effects of angiotensin 2 are well established, the specific effects of aldosterone on the vasculature are starting to be unraveled and are likely more relevant in the context of pathophysiology.

In this review, we will cover the basics of the RAAS proteolytic cascade, its regulation by various stimuli, and the major effects of angiotensin 2 and aldosterone, with a specific focus on volume homeostasis and vascular resistance. Aspects of the RAAS that are clearly of pathophysiologic relevance, such as the proliferative and profibrotic effects of angiotensin 2, are touched upon only marginally.

### The proteolytic cascade of the renin angiotensin aldosterone system

**Fig. 1:**
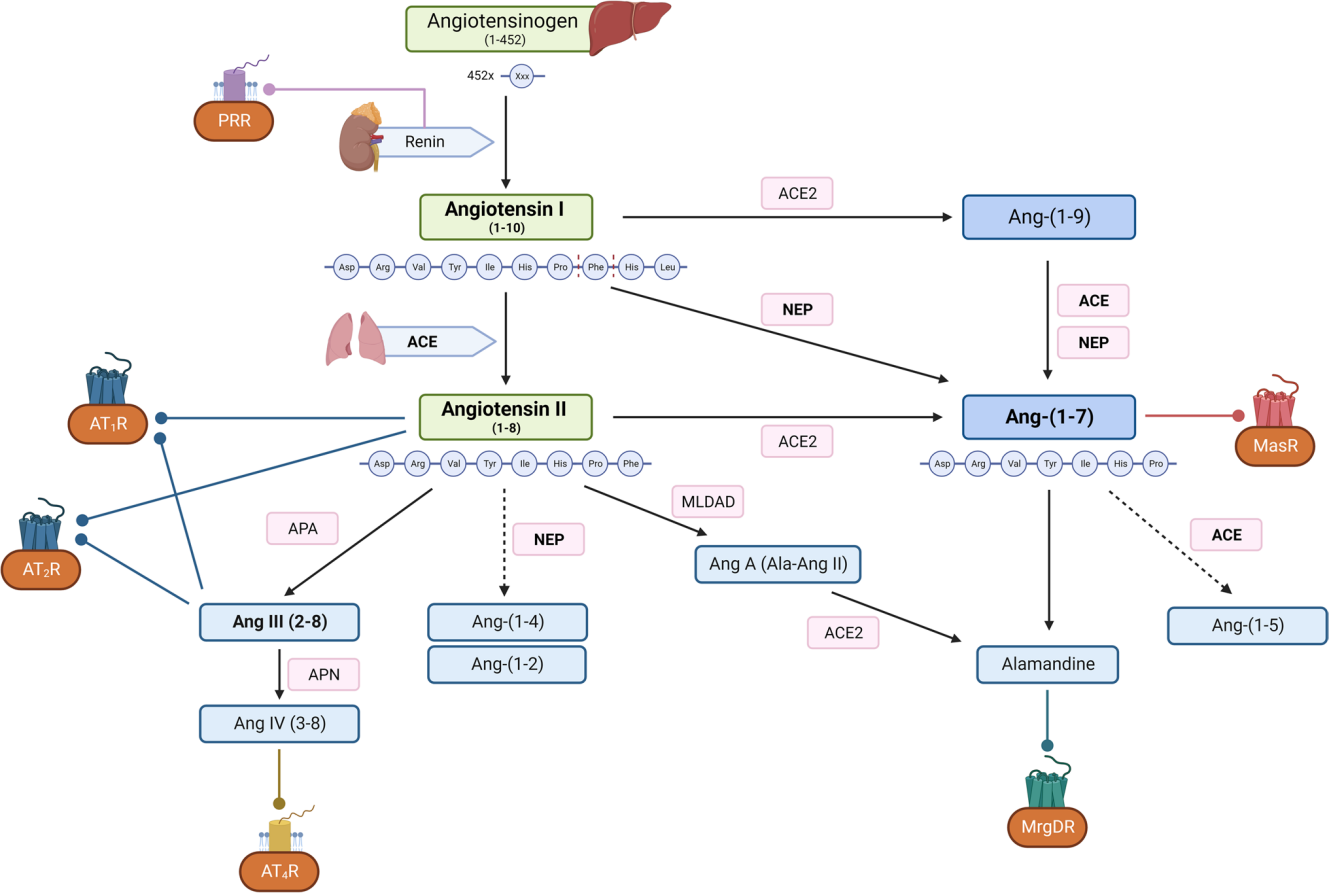
The proteolytic cascade of the RAAS. Renin released from the kidney cleaves the preprohormone angiotensinogen to form angiotensin I (Ang-(1–10)). Angiotensin I is subsequently cleaved by ACE activity to the active angiotensin II (Ang-(1–8)), acting on AT1 and AT2 receptors. Alternatively, angiotensin I is cleaved directly through NEP to Ang-(1–7), and through ACE 2 to Ang-(1–9), which is subsequently converted to Ang-(1–7). Ang-(1–7) mediates its effects by binding to the Mas receptor. It can be further cleaved by ACE to Ang-(1–5), and by decarboxylation to alamandine, a substrate of MrgDR. Further cleavage forms Ang-(1–7), Ang A, and alamandine. Ang II is also converted to Ang III. Ang III is the source of Ang IV, which binds to the AT4 receptor. ACE, angiotensin-converting enzyme; ACE2, angiotensin-converting enzyme type 2; NEP, neprilysin; Ang, angiotensin; APA, aminopeptidase A; APN, aminopeptidase N; MLDAD, mononuclear leukocyte-derived aspartate decarboxylase; PRR, prorenin receptor; AT_1_R, angiotensin II receptor type 1; AT_2_R, angiotensin II receptor type 2; AT_4_R, angiotensin II receptor type 4; MrgDR, Mas-related G protein–coupled receptor member D; MasR, Mas receptor

Angiotensin 2 and aldosterone are the RAAS effectors. Angiotensin 2 is generated in a proteolytic cascade that includes the cleavage of liver-derived angiotensinogen by the protease renin, producing the decapeptide angiotensin 1 (Fig. 1). In addition to catalyzing the rate-limiting step of the RAAS cascade, renin binds to the renin/prorenin receptor [2]. The binding of renin/prorenin to its receptor triggers a variety of cellular responses, the functional relevance of which is starting to be unraveled but is beyond the scope of this review. The dominant source of renin in the circulation is granulated renin-producing cells of the afferent arterioles of the kidney. Two amino acids are subsequently removed from angiotensin 1 by the activity of the angiotensin-converting enzyme (ACE), resulting in the octapeptide angiotensin 2. Angiotensin 2, in turn, stimulates the synthesis of aldosterone in the zone glomerulosa of the adrenal gland by promoting the activity of the steroidogenic acute regulatory (StAR) protein and aldosterone synthase.

Angiotensin 2 binds to AT1 and AT2 receptors. Most of the classic effects of angiotensin 2 are mediated by the G_q_-coupled AT1 receptor; the AT1-mediated effects of angiotensin 2 are partially counteracted or buffered by the AT2 receptor. More details on the function of AT1 and AT2 receptors are given below. The steroid hormone aldosterone, as the second endpoint of the RAAS, binds to the cytosolic mineralocorticoid receptor (MR), and, upon translocation of the aldosterone/MR complex to the nucleus, mediates predominantly genomic effects, i.e., modulates gene expression in target cells. In addition, recent data suggest that aldosterone exerts rapid, nongenomic effects with significant clinical implications. The nongenomic effects of aldosterone are not restricted to epithelial cells and include, but likely are not limited to, an increase in IP_3_ and diacylglycerol formation and an activation of protein kinase C [11, 34].

In addition to the classic effectors of the RAAS, angiotensin 1– and angiotensin 2–derived peptides are generated by various amino-, endo-, and carboxypeptidases. As shown in detail in Fig. 1, the products are different peptides, such as angiotensin 2–10, angiotensin 2–8, angiotensin 3–8, and angiotensin 1–7, along with their respective receptors, including MasR, AT_4_, and the Mas-related G protein–coupled receptor type D, MrgD. The function of these peptides is subject to intense research, because (i) they may account for previously unknown effects of the RAAS, and (ii) their formation is influenced in patients by commonly used ACE inhibitors (Fig. 1).

#### Beyond the endocrine system: ACE2 as the cellular entry site of respiratory viruses

The ACE2-ANG-(1–7)-Mas receptor axis constitutes a second branch of the classic RAS. Although various functions of this part of the RAS start to unravel, the presumably dominant function of the ACE2-ANG-(1–7)-Mas receptor axis consists of counteracting and finetuning the effects of the classic RAS. Furthermore, independent of its catalytic function, ACE2 is of major pathological relevance. Thus, ACE2 acts as the cellular receptor for several viruses, including the severe acute respiratory syndrome coronavirus (SARS-CoV), the human coronavirus-NL63, and, more recently, SARS-CoV-2, the virus accounting for the COVID-19 pandemic [12, 27, 57]. The broad expression pattern of ACE2 in humans likely is the reason why SARS-CoV-2 is a systemic rather than a respiratory disease, affecting virtually all organs of the body. Thus, in the respiratory system, which is the primary entry site of the virus, ACE2 is expressed in type I and type II alveolar epithelial cells of the lung, bronchiolar-epithelial cells, endothelial cells, and arterial smooth muscle cells of the entire respiratory system [21]. Furthermore, ACE2 expression is present in the heart, including endothelial cells and smooth muscle cells of coronary arteries and the intramyocardial vessels [6, 53]. ACE2 is also expressed in the kidney, testes, ovaries, liver, lung, intestine, and brain [21]. Consequently, ACE2 appears as a reasonable target for COVID-19 therapy and/or prevention. In principle, targeting ACE2 may include the application of (i) decoy ACE2 molecules to bind and neutralize the virus, of (ii) competitive pseudo ligands for ACE2, of (iii) agents that block the interaction of the Sars-CoV-2 spike protein and ACE2, and (iv) interventions to modulate the cell surface expression of ACE2 [23]. Importantly, the enzymatic activity of ACE2 is independent of binding of SARS-CoV-2 to ACE2, suggesting that blockade of the virus-binding site within the ACE2 protein may not have a major impact on the function of the ACE2-ANG-(1–7)-Mas receptor axis [15, 28]. The potentials and pitfalls of these strategies, all of which are currently experimental, were recently summarized in comprehensive reviews [23, 52].

In addition to the systemic RAAS, there are several local RASs consisting of all components that are necessary for the generation of angiotensin 2. Some of the local RASs, such as those in the brain and testis, are isolated from the systemic RAAS, as they operate beyond blood-tissue barriers [10]. The local RAS normally contribute little to the circulating levels of renin and angiotensin 2, but their effects may not be locally restricted to the originating tissue if the tissue is massively expanded, such as in the adipose tissue in obese individuals.

#### The renin/prorenin receptor

The activity of the local RASs, including the tissue-specific generation of angiotensin 2, are controlled by the local expression of the renin/prorenin receptor (PRR). The PRR was discovered as a single-transmembrane 45 kDa protein in the human kidney [38]. However, the following studies revealed that the expression of the PRR is not restricted to the kidney. Thus, PRR is present in the heart, testes, brain, placenta, thyroid and adrenal gland, and various cell types of the immune system [55]. The PRR has the following multifaceted functions: First, binding of renin to the receptor markedly increases its catalytical activity [38]. Second, binding of prorenin to the PRR leads to conformational changes of the prorenin protein, rendering prorenin catalytically active, and, eventually, leading to the enhanced local generation of angiotensin 1. Third, upon binding of prorenin, the PRR induces intracellular signaling pathways, like the ERK1/ERK2 and p38 MAP kinase pathway, which are relevant in the context of multiple cellular responses, such as apoptosis, formation of reactive oxygen species, and the enhanced formation of extracellular matrix proteins [44]. Forth, the PRR interacts with the Wnt receptors frizzled 8 and LRP6, facilitating Wnt signaling with multiple downstream effects, including cell survival and proliferation [14]. Fifth, through binding of its transmembrane carboxy-terminal subunit to the V-ATPase, PRR facilitates the assembly of the V-ATPase, which is required for the acidification of lysosomal vesicles. Additionally, the PRR may act as a pH-sensor and modify V-ATPase activity [26].

### Regulation of the RAAS

**Fig. 2:**
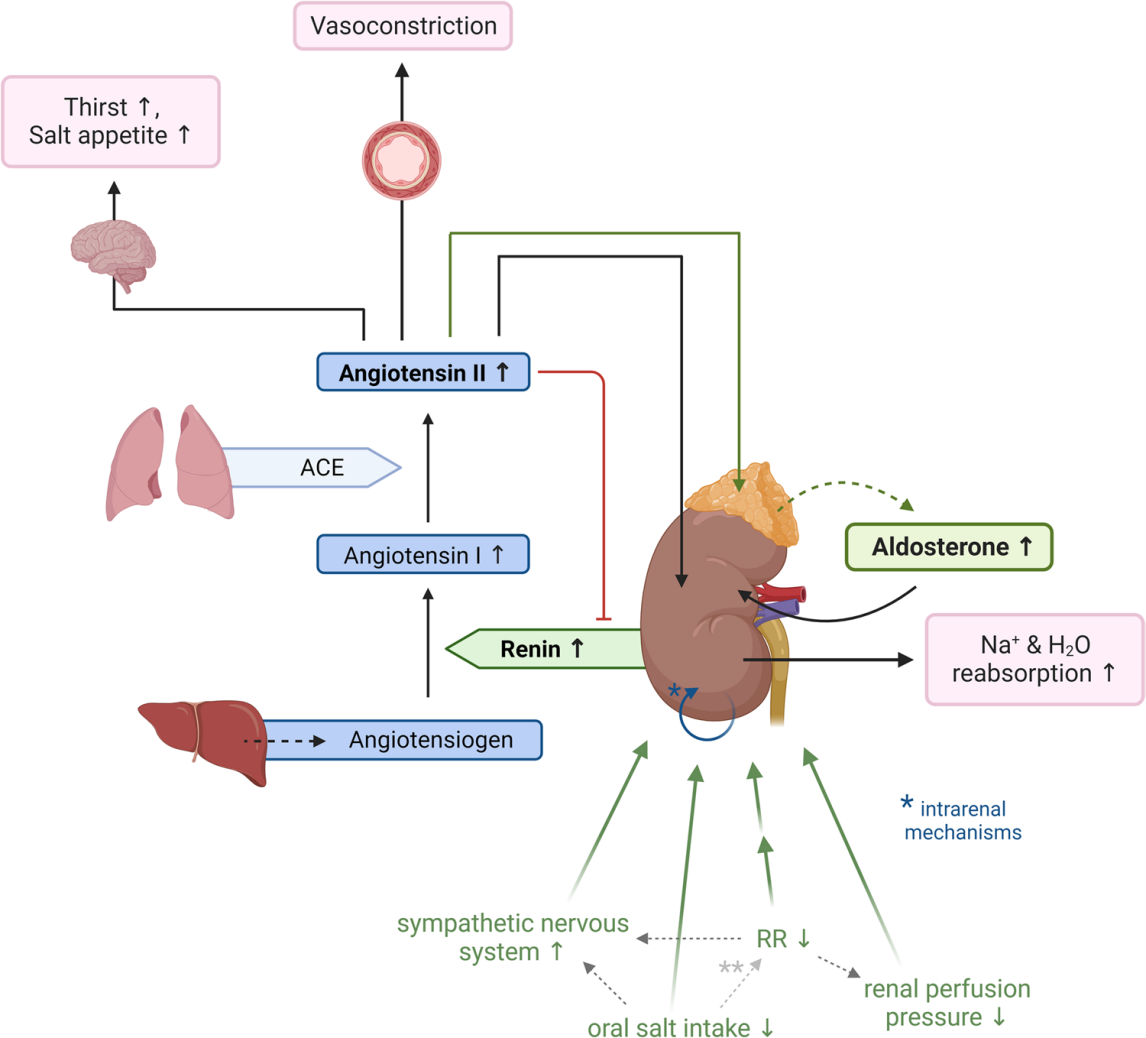
RAAS regulation and the cardinal effects of angiotensin 2 and aldosterone. The RAAS is regulated by a complex interplay of various parameters that operate to maintain the volume status and blood pressure. Normal arrow heads indicate stimulatory; block arrow heads indicate inhibitory pathways. Depicted is the stimulatory arm of the control of the RAAS. * indicates intrarenal mechanisms, such as the intrarenal baroreceptor and the macula densa mechanism of renin secretion. ** indicates the effect of oral salt intake on blood pressure which remains controversial and presumably is heterogenous (salt-sensitive and salt-resistant individuals)

Multiple regulatory mechanisms impinge on the RAAS and provide a regulatory network that ensures strict RAAS-dependent homeostasis of the volume status and blood pressure, as summarized in Fig. 2. Some of these regulatory mechanisms act in parallel; some act synergistically. In general, homeostatic regulatory networks, which are secured by multiple and interwoven mechanisms, have evolved for the maintenance of *critical* functions of the body. These include, but are not limited to, regulatory networks that provide control over parameters such as the body weight, growth, regeneration, and gonad function. A *critical* function, in this context, is a function that provides a considerable evolutionary advantage for an individual, and, consequently, is favored by selection processes. In terms of the conservation of such functions during evolutionary selection, it is important to consider that the decompensation of a function remains irrelevant, if it occurs after the reproductive phase of life. Thus, given that the RAAS has evolved to provide strict maintenance of blood pressure, its pathophysiologic impact, i.e., the development of hypertension and concomitant cardiovascular diseases, is not selected against during evolution, if such regulatory decompensation predominantly occurs in the postreproductive phase of life.

#### Pressure-dependent regulation of the RAAS

Because the RAAS serves to stabilize blood pressure, it is primarily regulated by parameters that change if blood pressure is outside the normal range (hypotension or hypertension). In this context, the most important stimulus of renin secretion is probably provided by the sympathetic nervous system acting on ß1 receptors of the renin-producing cells. Thus, in ß1 receptor–deficient mice, the baseline plasma renin concentration (PRC) is reduced by approximately 85% [25]. The sympathetic nervous system receives information about blood pressure via baroreceptors, which are localized in the aortic arch and carotid sinus. Afferent signals reach the medullary cardiovascular center via vagal and glossopharyngeal fibers. Experiments in the isolated perfused kidney suggest that, in addition to being regulated by systemic blood pressure, renin secretion is also determined by an intrarenal baroreceptor mechanism. This mechanism presumably consists of 2 components: (i) pressure-dependent glomerular filtration and proximal tubular salt reabsorption, which modulate the NaCl concentration at the macula densa segment of the thick ascending limb of Henle’s loop, and (ii) a poorly defined intrarenal baroreceptor, located in the afferent arteriole and/or the renin-producing cells. First, the macula densa mechanism is activated by changes in the luminal Cl^−^ concentration at the macula densa segment, which occur if the GFR changes and/or if salt and water reabsorption in segments of the nephron upstream of the macula densa is altered [50]. Subsequently, changes in macula densa salt transport via NKCC2 are translated into the release of paracrine factors, which eventually modulate renin secretion. The stimulatory axis of the macula densa is activated when the tubular Cl^−^ concentration is low and predominantly leads to the release of prostaglandin E2 acting on EP receptors on renin-producing cells [50]. Conversely, macula densa–dependent inhibition of renin release is triggered by an increase in the tubular Cl^−^ concentration and results in the release of ATP, which is readily degraded to adenosine in the confines of the juxtaglomerular apparatus. Adenosine acts on A1A receptors of renin-producing cells and suppresses renin secretion [10, 49]. Second, the afferent arteriolar baroreceptor, which is located in, or adjacent to, renin-producing cells. This local baroreceptor is responsive to changes in pressure rather than flow [37]. This finding appears reasonable, because an (pressure-dependent) increase in flow would be expected to facilitate endothelium-derived NO formation, a stimulator of renin secretion [10], whereas increases in perfusion pressure suppress renin secretion in the isolated kidney [49]. Despite intense research efforts, the local baroreceptor in the afferent arteriole remains poorly defined. A new hint to understand the yet cryptic local baroreceptor may be the discovery of mechanosensitive Na^+^-channels of the Piezo2 type in renin-producing cells [36]. The activation of Piezo2 by mechanical stress is expected to depolarize renin-producing cells, leading to the inhibition of renin secretion. Commensurate with this assumption, the resting membrane potential of renin-producing cells in nonperfused arterioles is in the range of − 70 mV and depolarizes to − 40 mV in arterioles perfused with a constant pressure of 100 mm Hg [31]. However, at the cellular level, the link between membrane depolarization and the inhibition of renin release remains unclear. Thus, the activation of l-type voltage-dependent Ca^2+^ channels during depolarization may mediate an influx of Ca^2+^, and subsequently inhibit renin release [10]. However, the functional relevance of l-type voltage–dependent Ca^2+^ channels in renin-producing cells remains controversial [19, 48].

The inhibition of renin secretion in response to increased renal perfusion pressure may also be detected by cells in the vicinity of renin-producing cells and then be propagated from adjacent cells to the renin-producing cells, as uncoupling of renin-producing cells by genetic deletion of connexins, specifically connexin 40, renders the RAS nonresponsive to local pressure changes [54].

#### Ang II feedback loops

Like in many other endocrine systems, angiotensin 2 inhibits its own production via direct and indirect effects on renin secretion via feedback loops. The negative feedback loop of angiotensin 2 on renin secretion consists of (i) an indirect (“long”) feedback loop, which is mediated by its systemic, blood pressure increasing effect, and (ii) a direct (“short”) feedback loop, mediated by the activation of AT1 receptors on renin-producing cells of the afferent arterioles [7]. The existence of the latter is supported by the finding that renin secretion in response to angiotensin 2 infusions is suppressed, even if the dosage of angiotensin 2 is below the threshold for pressure responses [16]. Similarly, in the isolated perfused kidney, at constant perfusion pressure, renin secretion is suppressed in response to angiotensin 2 [45]. Finally, angiotensin 2 inhibits the release of paracrine factors that simulate renin secretion. For example, the expression of cyclooxygenase-2 in the macula densa of the mouse is downregulated by angiotensin 2 administered in subpressor doses, limiting the synthesis of renin-stimulatory prostanoids [56]. Similarly, macula densa cells produce NO catalyzed by neuronal nitric oxide synthase (nNOS), which stimulates renin secretion. The expression of nNOS in macula densa cells, again, is suppressed by angiotensin 2 [3].

#### The link between oral salt intake and RAAS regulation

It has been known for decades that high dietary salt intake suppresses the RAAS, whereas oral salt restriction is a potent stimulator of the RAAS [10]. Although the connection between salt intake and RAAS activity is well established, the mechanistic link remains enigmatic. There is experimental evidence for several factors that may contribute to the salt-dependent regulation of the RAAS. However, when reviewed critically, many of the results appear conflicting and fail to provide a coherent picture. Oral salt intake may influence the RAAS by factors including changes in blood pressure, renal nerve activity, the macula densa mechanism, and a variety of humoral factors [4, 10, 41].

### Effects of the RAAS: volume homeostasis

As mentioned above, the long-term homoeostasis of the volume status, as a parameter that directly influences blood pressure, is regulated by a balanced intake of salt and water and the concomitant excretion of the same amount of salt and water over time. Perturbations of the volume status are therefore compensated by adaptations of salt/water intake and/or by changes in renal excretion. The RAAS influences both parameters.

#### Angiotensin 2 and aldosterone: renal salt conservation

Angiotensin 2 reduces renal salt loss by (i) reducing the GFR and (ii) stimulating salt reabsorption along the nephron. First, AT1 receptors in the renal vasculature are expressed in the afferent and efferent arterioles. There has been some discussion as to whether angiotensin 2 has a more pronounced effect on the afferent or efferent arteriole. An isolated angiotensin 2–mediated constriction of the efferent arteriole may increase the GFR if total renal blood flow remains stable. Conversely, the preferential constriction of the afferent arteriole is expected to lower GFR, and the same is the case if the afferent and efferent arterioles constrict to a similar degree. Most data obtained in vivo, in the isolated perfused kidney, in micropuncture experiments, and in the isolated perfused juxtaglomerular apparatus preparation, however, suggest that the net effect of angiotensin 2 on the GFR is inhibitory [4]. Second, regarding Na^+^ tubular handling, angiotensin 2 activates various salt reabsorptive transport systems along the tubular system mediated by AT1 receptors. Thus, angiotensin 2 enhances NHE3, NHE1, ENaC β, NKCC2, and NCC protein expression [30]. Angiotensin 2 also increases the *activity* of the NaCl cotransporter (NCC) through a WNK4-SPAK-dependent pathway [43].

The promotion of renal salt reabsorption by angiotensin 2 is further supported by aldosterone. Thus, aldosterone increases sodium reabsorption in the distal nephron and the collecting duct. The primary targets are NCC and eNaC. For NCC, the stimulatory effect is mediated by the increased expression of SGK1, which phosphorylates NEDD4-2, leading to reduced proteasomal degradation of WNK1. WNK1 eventually phosphorylates NCC to activate the NCC [9]. In the principal cells of the collecting ducts, aldosterone binds to the MR and induces the expression of eNaC subunits, accompanied by an increased expression of the basolateral Na/K-ATPase and components of the respiratory chain of the mitochondria [5]. Consequently, aldosterone impinges on the entire Na^+^ uptake machinery of the collecting duct principal cells.

#### Thirst and salt appetite: behavioral responses to changes in angiotensin 2 and aldosterone plasma concentrations

The most straightforward strategy to overcome hypovolemia, which may or may not be accompanied by extracellular hyperosmolarity, is the induction of thirst and subsequent drinking behavior. Given that most natural fluids are hypotonic compared to plasma, drinking hypotonic fluids (water) will compensate for hypovolemia and normalize extracellular hyperosmolarity. For isotonic hypovolemia (e.g., because of blood loss) drinking water restores the volume status but comes at the price of dilution-induced hypoosmolarity. Data from studies in animals and humans suggest that systemic infusions of angiotensin 2 in high concentrations elicit thirst, and, consequently, the initiation of drinking behavior. For example, in rats, the threshold for a dipsogenic response was reached at a plasma angiotensin 2 concentration of 460 pg/mL, a concentration similar to what was seen after 48 h of water restriction [1]. The high threshold for angiotensin 2 to induce thirst leads to questions regarding the major relevance of systemic angiotensin 2 in normal drinking behavior. Thus, more robust and pronounced dipsogenic responses were induced by intracranial infusions of angiotensin 2, suggesting that angiotensin 2 generated by the local brain RAS may be dominant in eliciting thirst [1]. Following the dipsogenic response with some delay, angiotensin 2 elicits increased salt appetite. The increased oral salt intake, in parallel with renal salt conservation, is needed for the normalization of isotonic hypovolemia (e.g., after blood loss) if the induced drinking behavior adds hypotonic volume to the system, which is usually the case. The reason for the apparent different kinetics of induction of thirst (fast) and salt appetite (slow) by angiotensin 2 is not entirely clear, but angiotensin 2 may act in part indirectly, via the generation of aldosterone. Thus, aldosterone or deoxycorticosterone acetate (DOCA) infusion in rats has been shown to induce salt appetite via central effects, despite a concomitant suppression of renin secretion and renal salt retention [18, 42].

#### Can volume homeostasis and the control of extracellular K+ concentration be separated from each other?

As outlined above, hypovolemia and concomitant hypotension activate the RAAS through multiple pathways. The main effectors of the RAAS cascade, angiotensin 2 and aldosterone, work in concert to restore the volume status and to normalize blood pressure. As a side effect of this compensatory response to hypovolemia, increased concentrations of aldosterone inevitably impinge on K^+^ homeostasis. Thus, Na^+^ reabsorption and K^+^ secretion are functionally linked in the aldosterone-sensitive portions of the tubular system and the collecting duct. The aldosterone-induced reabsorption of Na^+^ via eNaC (see above) inevitably leads to K^+^ secretion driven by changes in the cellular membrane potential, and therefore eventually causes renal K^+^ loss. However, K^+^ secretion in the aldosterone-sensitive portion of the nephron is also a function of luminal flow. Thus, at low luminal flow, K^+^ secretion is low in the collecting duct for a given aldosterone concentration. Adequate flow and the washout of K^+^ from the surface of collecting duct principal cells are prerequisites for membrane potential-driven K^+^ secretion. Angiotensin 2 decreases GFR and stimulates salt and water reabsorption in portions of the nephron upstream of the collecting ducts. Both effects lead to lower flow rates in the collecting duct and, consequently, buffer the direct effects of aldosterone on K^+^ secretion. In addition, in humans, the slope of plasma aldosterone concentration versus plasma K^+^ is steeper compared to the slope of plasma aldosterone versus plasma angiotensin 2, at least in the normal range of 3.5–5.2 mM K^+^ and 10–50 pM angiotensin 2 [47], respectively [17, 58]. The correlation suggests that, if the system does not exceed the normal concentrations of K^+^ and angiotensin 2, plasma aldosterone concentration is predominantly determined by plasma K^+^. However, in situations of excessively high aldosterone concentrations, such as after massive blood loss, the diuretics use, salt-losing nephropathies of various etiologies, and hyperaldosteronism, aldosterone-induced hypokalemia will occur.

High K^+^ ingestion, in turn, stimulates aldosterone production independent of angiotensin 2 [40]. Potassium added to the system by food inevitably leads to transient hyperkalemia. As a first line defense, ingested K^+^ is rapidly shifted from the extracellular to the intracellular compartment by the action of insulin. The long-term correction of total potassium content, however, requires the balanced renal elimination of surplus K^+^. Thus, the aldosterone generation induced by hyperkalemia facilitates renal K^+^ secretion. At first glance, by promoting renal K^+^ elimination, concomitant eNaC-dependent Na^+^ reabsorption will lead to Na^+^ retention and, potentially, an increase in blood pressure. However, most epidemiologic studies suggest that dietary K^+^ is inversely related to blood pressure and the incidence of cardiovascular diseases if K^+^ is not excessively ingested [8, 24]. Thus, the K^+^-induced generation of aldosterone is compensated for by other, blood pressure-lowering mechanisms. In fact, hyperkalemia was shown to reduce the tone of the vascular smooth muscle cells of resistance vessels and dampen sympathetic nerve activity [20]. In the kidney, high plasma K^+^ inhibits NCC activity in the distal convoluted tubule (DCT), which may counteract or even cancel out the anti-natriuretic effects of aldosterone on NCC and eNaC [35]. In fact, NCC is rapidly dephosphorylated in response to oral K^+^ intake, reducing its transport activity [51].

### Effects of the RAAS: vascular resistance

#### Vascular effects of angiotensin 2

As mentioned above, angiotensin 2 is one of the most potent vasoconstrictors of the body. Its constrictor effect is mediated through the activation of G_q_-coupled AT1 receptors, which are abundantly expressed in the smooth muscle cells of the vasculature. In contrast to the AT1 receptor, the signaling linked to AT2 is more variable and depends on the cell type. AT2-mediated intracellular signaling includes, but is not limited to, G_i_-coupled signaling, ERK pathway inhibition or activation, protein phosphatase 2A, and NO/cGMP pathway regulation [39]. The latter buffers the effects of angiotensin 2 on AT1 receptors to (locally) attenuate vasoconstriction [22]. In addition to vascular effects, AT2 receptor activation is blood pressure lowering through its negative chronotropic effect on the heart, as shown by the cardiac-specific overexpression of the AT2 receptor in mice [33].

#### Vascular effects of aldosterone

Aldosterone has a variety of fast and nongenomic effects on the vasculature that are just about to be unraveled. These effects comprise the dilatory actions of aldosterone mediated via the endothelium (e.g., the generation of nitric oxide) and direct constrictor effects on vascular smooth muscle cells [13]. The latter is, at least in part, mediated by the newly discovered G protein–coupled estrogen receptor (GPER) [29]. The net effect of aldosterone on vascular resistance depends on the location of the specific vessel, the concentration of aldosterone, and the exposure time [32]. The nongenomic effects of aldosterone on vascular resistance are potential therapeutic targets because chronic pharmacologic blockade of the RAAS is accompanied by increases in aldosterone plasma concentrations in patients (the so-called aldosterone breakthrough phenomenon [46]), and chronically elevated aldosterone levels shift the balance between vasodilator and vasoconstrictor effects of aldosterone in favor of vasoconstriction [13]. Furthermore, recent data suggest that excess aldosterone is detrimental to the endothelium including the endothelial glycocalyx, leading to reduced NO formation in response to shear stress, and, consequently, compromised vasodilatory capacity [13].

## Summary

The RAAS primarily evolved as an endocrine system that facilitates salt/water homeostasis and the control of vascular resistance, the central parameters of the regulation of arterial blood pressure. This function of the RAAS is mediated primarily by angiotensin 2 and aldosterone. Recent data, however, suggest that multiple additional sidechains of the RAAS modify and fine-tune the effects of the classical RAAS. These include several angiotensin 1– and 2–derived peptides with respective receptors, such as the MasR, AT_4_, and MrgD receptors. Furthermore, renin binds to the PRR, inducing changes in renin/prorenin activity and triggering multiple intracellular signaling pathways. Finally, as a pathological aspect, which is independent of any endocrine RAAS function, the broad expression pattern of components of the RAAS, specifically of ACE2, provides the cellular entry pathway for several viruses, most notably that of SARS-CoV-2.

## Data Availability

Not applicable.
